# Self-administration of injectable contraception: a systematic review and meta-analysis

**DOI:** 10.1136/bmjgh-2018-001350

**Published:** 2019-04-02

**Authors:** Caitlin E Kennedy, Ping Teresa Yeh, Mary Lyn Gaffield, Martha Brady, Manjulaa Narasimhan

**Affiliations:** 1 Johns Hopkins University Bloomberg School of Public Health, Baltimore, Maryland, USA; 2 Department of International Health, Johns Hopkins University Bloomberg School of Public Health, Baltimore, Maryland, USA; 3 Human Reproduction Programme (HRP), Organisation mondiale de la Sante, Geneve, Switzerland; 4 PATH, Washington, District of Columbia, USA; 5 Human Reproduction Programme (HRP), World Health Organization, Geneva, Switzerland

**Keywords:** self-administration, injectable contraceptive, DMPA, systematic review, meta-analysis

## Abstract

**Introduction:**

Depot medroxyprogesterone acetate subcutaneous injectable contraception (DMPA-SC) may facilitate self-administration and expand contraceptive access. To inform WHO guidelines on self-care interventions, we conducted a systematic review and meta-analysis comparing self-administration versus provider administration of injectable contraception on outcomes of pregnancy, side effects/adverse events, contraceptive uptake, contraceptive continuation, self-efficacy/empowerment and social harms.

**Methods:**

We searched PubMed, Cumulative Index to Nursing and Allied Health Literature, LILACS and EMBASE in September 2018 for peer-reviewed studies comparing women who received injectable contraception with the option of self-administration with women who received provider-administered injectable contraception on at least one outcome of interest. Risk of bias was assessed using the Cochrane tool for randomised controlled trials (RCTs) and the Evidence Project tool for non-randomised studies. Meta-analysis was conducted using random-effects models to generate pooled estimates of relative risk (RR).

**Results:**

Six studies with 3851 total participants met the inclusion criteria: three RCTs and three controlled cohort studies. All studies examined self-injection of DMPA-SC; comparison groups were either provider-administered DMPA-SC or provider-administered intramuscular DMPA. All studies followed women through 12 months of contraceptive coverage and measured (dis)continuation of injectable contraception. Meta-analysis found higher rates of continuation with self-administration compared with provider administration in three RCTs (RR: 1.27, 95% CI 1.16 to 1.39) and three controlled cohort studies (RR: 1.18, 95% CI 1.10 to 1.26). Four studies reported pregnancies; all showed no difference across study arms. Four studies reported side effects/adverse events; while two controlled cohort studies showed increased injection site reactions with self-administration, no other side effects increased with self-administration. One study found no difference in social harms. No studies reported measuring uptake or self-efficacy/empowerment.

**Conclusion:**

A growing evidence base suggests that self-administration of DMPA-SC can equal or improve contraceptive continuation rates compared with provider administration. This benefit comes without notable increases in pregnancy or safety concerns. Self-injection of DMPA-SC is a promising approach to increasing contraceptive use.

**Key questions**

**What is already known?**

- New subcutaneous forms of injectable contraception may allow women to self-inject contraception more easily without requiring a provider, potentially expanding access to this safe and efficacious contraceptive method.

**What are the new findings?**

- A systematic review of the literature identified six studies from a range of country settings.
- In the meta-analysis, the relative risk of contraceptive continuation was higher with self-administration of injectable contraception compared with provider administration.
- There were no major differences in pregnancy or side effects/adverse events, except that the two controlled cohort studies showed increased injection site reactions with self-administration.

**What do the new findings imply?**

- A small but growing evidence base provides consistent evidence to suggest that self-administration of depot medroxyprogesterone acetate subcutaneous injectable contraception can lead to improved contraceptive continuation rates and equivalent pregnancy prevention compared with provider administration.

## Introduction

Self-care in the context of sexual and reproductive health and rights (SRHR) occurs when individuals ‘are able to identify their own health needs, to access appropriate health technologies, and to effectively manage their health conditions—including seeking health services and professional help when necessary’.^1^ Self-care in SRHR includes the ability of women of reproductive age to assess and manage their own fertility needs.^2^ Depot medroxyprogesterone acetate (DMPA), a progestogen-only contraceptive injectable, is widely used by women around the world. In recent years, a subcutaneous product (DMPA-SC) has been developed that can be injected relatively easily compared with intramuscular products (DMPA-IM) and that has been found safe and efficacious.^3 4^ This subcutaneous product is being produced and marketed as a prefilled needle and drug combination, which is now available in at least 20 Family Planning 2020 (FP2020) countries and has regulatory approval in over 40 countries.^5 6^ Allowing women to self-inject DMPA may remove barriers to DMPA continuation, specifically challenges around returning to a healthcare facility every 3 months for a new injection. There is hope that self-injection of DMPA-SC, or other injectable contraceptives, could expand access to contraception for women facing challenges to regular access to healthcare settings or where provider shortages limit availability.

In 2017, Kim *et al* 7 published a systematic review which found that, compared with provider administration of injectable contraception, self-administration showed little to no difference in continuation rates and no indication of safety concerns (as there were no adverse events). The review concluded that ‘with appropriate information and training the provision of contraceptive injectables for the woman to self-administer at home can be an option in some contexts’.^7^ However, the review included studies published through October 2015, and there have been several recent publications in this area. To inform forthcoming WHO guideline on self-care interventions for SRHR, we sought to update the review by Kim *et al* 7 by conducting a systematic review of the evidence for provision of injectable contraception using self-administration compared with provider administration.

## Methods

### PICO question and inclusion criteria

This review followed the PICO (population, intervention, comparison and outcomes) question: Should self-administration be made available as an additional approach to deliver injectable contraception?

- Population: individuals of reproductive age using injectable contraception.
- Intervention: provision of injectable contraception including self-administration options.
- Comparison: provision of injectable contraception that does not include self-administration as an option (provider-administered only).
- Outcomes: (1) pregnancy; (2) side effects or adverse events (eg, bleeding, skin site reactions, mental health); (3) uptake of injectable contraception (initial use); (4) continuation rate of injectable contraception (or, conversely, discontinuation); (5) self-efficacy, knowledge and empowerment; and (6) social harms (eg, coercion, violence [including intimate partner violence, violence from family members or community members and so on], psychosocial harm, self-harm and so on), and whether these harms were corrected/had redress available.

To be included in the review, an article had to meet the following criteria:

- Study design that compares people who received injectable contraception that included the option of self-administration with people who received injectable contraception without an option for self-administration.
- Measured one or more of the outcomes listed above.
- Published in a peer-reviewed journal.

Where data were available, we stratified all analyses by the following subcategories:

- Type of support offered to self-administration.
- Age: adolescents and young people (ages 10–14, 15–19 and 15–24) and adults (ages 25+).
- Vulnerabilities (in relation to poverty, disability, living with HIV, sex work or literacy).
- High-income versus low-income or middle-income countries.

No restrictions were placed based on location of the intervention or language of the article. We planned to translate articles in languages other than English if identified.

### Search

The following electronic databases were searched through the search date of 5 September 2018: PubMed, Cumulative Index to Nursing and Allied Health Literature, LILACS (Latin American and Caribbean Health Sciences Literature) and EMBASE. We also searched the included articles from the previous review conducted by Kim *et al* 7 and from a 2018 special supplement on DMPA-SC in the *Contraception* journal. Secondary reference searching was conducted on all studies included in the review. Further, selected experts in the field were contacted to identify additional articles not identified through other search methods. Finally, we searched for ongoing trials through ClinicalTrials.gov, WHO International Trial Clinical Registry Platform (ICTRP), Pan African Clinical Trial Registry (PACTR) and Australian New Zealand Clinical Trials Registry.

The following search strategy was developed for PubMed and adapted for entry into all computer databases; a full list of search terms for all databases is available from the authors on request.

(“Sayana Press” [tiab] OR “depot medroxyprogesterone acetate” [tiab] OR “depo-medroxyprogesterone acetate” [tiab] OR “Depo Medroxyprogesterone Acetate” [tiab] OR “Medroxyprogesterone” [tiab] OR “Medroxyprogesterone Acetate” [tiab] OR DMPA [tiab] OR DMPA-SC [tiab] OR Uniject [tiab] OR Depo-Provera [tiab] OR “Depo Provera” [tiab] OR “Depo-Subq Provera” [tiab] OR “Long-Acting Reversible Contraception” [Mesh]) AND (self-administration [tiab] OR self-administer [tiab] OR self-administered [tiab] OR self-injection [tiab] OR self-inject [tiab] OR self-injected [tiab] OR “home use” [tiab] OR “home administration” [tiab] OR “home injection” [tiab] OR “self- vs provider-administered” [tiab] OR “self- and provider-administered” [tiab] OR “self- vs physician-administered” [tiab] OR “self- and physician-administered” [tiab] OR “self and clinical administration” [tiab] OR “self- vs clinician-administered” [tiab] OR “self and clinician administered” [tiab] OR “self care” [Mesh] OR self-administration [Mesh] OR self-assessment [Mesh])

Titles, abstracts, citation information and descriptor terms of citations identified through the search strategy were initially screened by a member of the senior study staff (CEK). The remaining citations were then screened in duplicate by two reviewers (CEK and PTY), with differences resolved through consensus. Final inclusion was determined after full-text review.

### Data extraction and analysis

For each included article, data were extracted independently by two reviewers using standardised data extraction forms. Differences in data extraction were resolved through consensus. Data extraction forms covered the following categories:

- Study identification: author(s), type of citation and year of publication.
- Study description: study objectives, location, population characteristics, type of injectable contraception (eg, DMPA), type of injection (eg, intramuscular, subcutaneous), injection site (eg, home, clinic), description of any additional intervention components (eg, any education, training, support provided), study design, sample size, follow-up periods and loss to follow-up.
- Outcomes: analytic approach, outcome measures, comparison groups, effect sizes, CIs, significance levels, conclusions and limitations.
- Risk of bias: assessed for randomised controlled trials (RCTs) with the Cochrane Collaboration’s tool for assessing risk of bias,^8^ and for non-randomised trials but comparative studies with the Evidence Project risk of bias tool.^9^

Where multiple studies reported the same outcome, we conducted meta-analysis using random-effects models to generate pooled relative risk (RR) using the program Comprehensive Meta-Analysis.^10^ Heterogeneity was assessed using both Q and I-squared statistics, and funnel plots were created to examine the potential for publication bias. Data from RCTs and non-randomised studies were analysed separately. When studies presented both strict and lenient definitions of continuation, taking into account injections that occurred just before or just after the reinjection window for example, we used the strict definition in meta-analysis. For studies where there were zero events in one study arm, we employed a continuity correction by adding 0.5 events to both study arms.

### Patient and public involvement

A representative of a patient group was invited to review both the protocol and final review. Patients and the public are currently involved in a global survey of values and preferences and in focus group discussions with vulnerable communities conducted to inform the WHO self-care guideline, and thus play a significant role in the overall recommendation outcome from this review.

## Results

### Description of included studies

**Figure 1:**
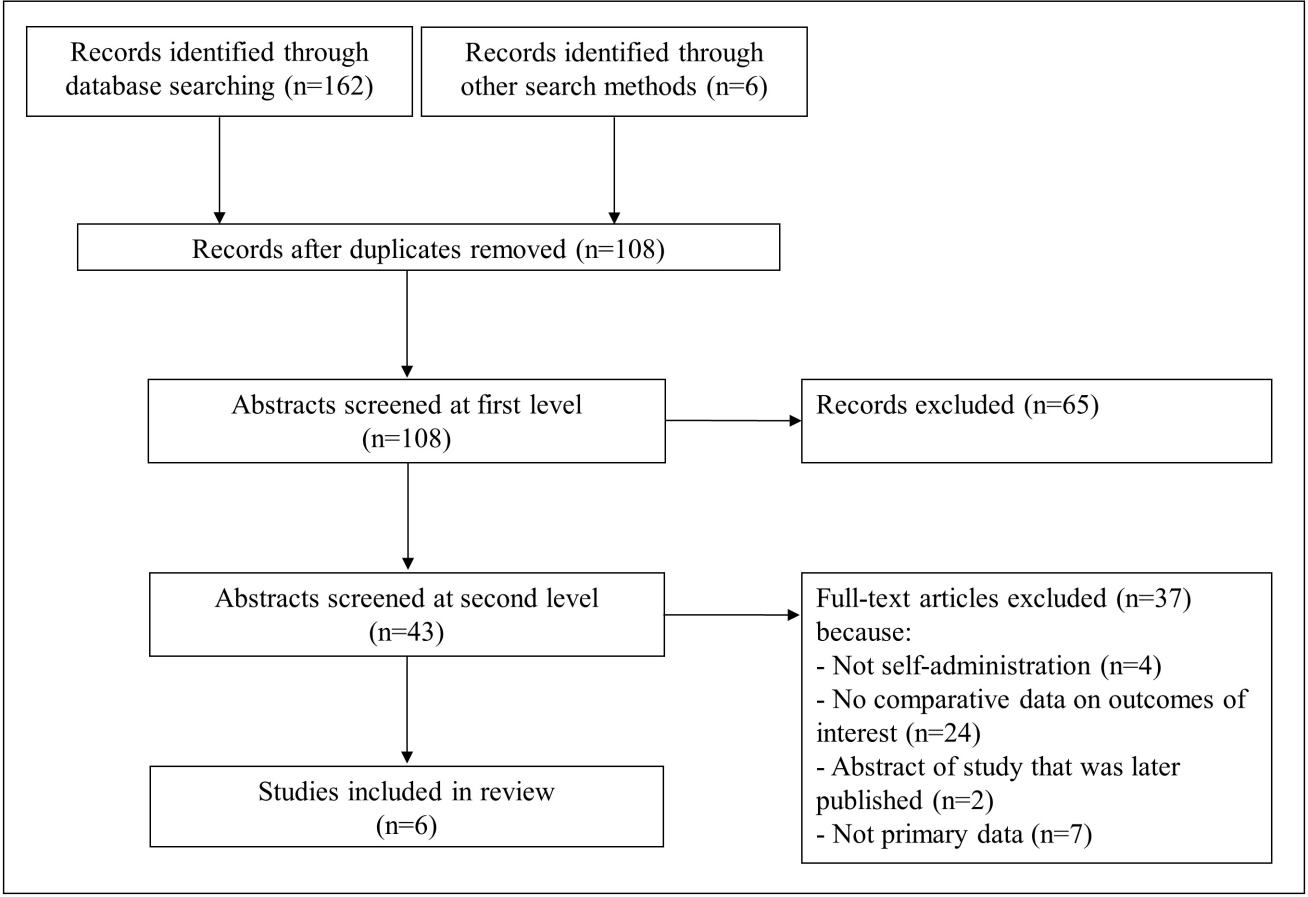
PRISMA flow chart showing disposition of citations through the search and screening process. PRISMA, Preferred Reporting Items for Systematic Reviews and Meta-Analyses.

Figure 1 presents a flow chart showing the study selection. The initial database search yielded 162 records, with 6 records identified through other sources; 108 remained after removing duplicates. After the initial title/abstract review, 43 articles were retained for full-text screening. Ultimately, six articles met the inclusion criteria and were included in the review.^5 11–15^ One article published in 2006—a prospective cohort trial with crossover among 16 participants—was reviewed carefully but ultimately not included in the review.^16^ Although it followed participants for contraceptive continuation over the course of the trial and measured pain, it did not present quantitative outcome data stratified by self-administration or provider administration. As a result, and because the study was significantly different from the other included articles due to a much smaller number of participants and a different study design, we have not included it in this review. However, we note that the study did report that 10 of 16 participants completed the full study protocol (including both self-administration and provider administration phases), and that there was no significant association between the type of injection and pain over the course of the study.

Table 1 shows the characteristics of the six included studies. The studies were published between 2012 and 2019 and included 3851 total participants; individual study sample sizes ranged from 128 to 1299. Locations included the USA (n=2), Scotland, Uganda, Senegal and Malawi. All studies included a study arm where women self-injected DMPA-SC. However, in three studies the comparison arm was provider-administered DMPA-SC, while in the other three studies the comparison arm was provider-administered DMPA-IM. Some studies provided reminder emails or text messages, either just to the self-injection arm participants or to all participants, while others provided calendar reminders at the first visit or no reminders other than the usual procedures. All studies had women either self-inject or return for provider-administered injections every 3 months (12–13 weeks), with some window for early and late injections, and all studies followed women through 12 months of contraceptive coverage.

**Table 1:** Study descriptions

Citation	Study location and population characteristics	Study design	Intervention description	Comparison group description
Beasley *et al* 11	New York City, USA. Women aged 18+ seeking DMPA for contraception. Mean age (±SD): (I) 26.0±6.1; (C) 26.1±6.3.	Randomised controlled trial. 132 participants (86 self-administration, 46 clinic administration). 12-month follow-up.	Self-administration of DMPA-SC. Participants were taught to self-inject Depo-SubQ Provera 104 by the study coordinator. The participant performed the initial injection in the abdomen or thigh under supervision, and if deemed acceptable was given prepackaged DMPA-SC containing a prefilled syringe and needle to use at home, along with alcohol pads, a bandage, a urine pregnancy test and a calendar with dates for the next injection. Each participant received a sharps disposal canister and instructions on safe needle disposal. At the 6-month visit, those in the self-administration group were re-evaluated for ability to self-inject and received additional prepackaged DMPA-SC for home administration.	Clinic administration of DMPA-SC. Participants received routine appointments for their next injections (every 3 months).
Burke *et al* 12	Mangochi District, Malawi. Women aged 18–40 years old receiving family planning services. Mean age: 26.9 years (SD: 5.21).	Randomised controlled trial. 731 participants (364 self-administration, 367 provider administration). 12-month follow-up.	Self-administration of DMPA-SC. All women received DMPA-SC injections in the form of Sayana Press 104 mg in a 0.65 mL suspension. Women who successfully self-injected at enrolment were given three doses of DMPA-SC to take home for subsequent self-injections and written instructions to remind them of the injection procedures. Participants could also ask the provider to train a trusted person to give them DMPA-SC at home. At enrolment, women were provided with a written note showing their future injection dates (every 13 weeks) and a calendar to assist them in remembering when to reinject.	Provider administration of DMPA-SC. At enrolment, women were provided with a written note showing their future injection dates (every 13 weeks) and a calendar to assist them in remembering when to return for reinjection.
Cameron *et al*, 2012^13^	Edinburgh, Scotland. Women aged 18–40 years old attending family planning clinic and existing users of DMPA-IM for at least 9 months. Mean age: (I) 29.2 years (SD: 5/0); (C) 28.8 (SD: 5.6).	Controlled cohort study. 128 participants (64 self-administration, 64 provider administration). 12-month follow-up.	Self-administration of DMPA-SC. Women were taught how to give a subcutaneous injection (Sayana) by the study research nurse. When deemed competent in the technique, they gave themselves the initial self-injection of DMPA-SC into their abdomen under nurse supervision at the clinic. Women were provided with three prefilled syringes of DMPA-SC, together with needles for subcutaneous injection, to take home and a list of dates when the three injections would be due. Women were also given a pack containing written information on the method of self-injection, advice on safe storage of the medicine and safe disposal of the needles (including a small ‘sharps’ disposal box) and 24-hour contact telephone numbers for advice. Women were sent a text message 1 week prior to the scheduled date of injection to remind them when the next injection was due.	Provider administration of DMPA-IM. No reminders were sent.
Cover *et al* 15	5 districts in Uganda. Women aged 18–45 years old attending participating health facilities for routine FP visits who expressed an interest in using injectable contraception (whether new, continuing or past injectable users). Mean age: (I) 26.9 (SD: 6.4); (C) 26.5 (SD: 6.2).	Controlled cohort study. 1161 participants (561 self-administration, 600 provider administration). 12-month follow-up.	Self-administration of DMPA-SC. Those who opted for self-injection were trained one-on-one and administered their first injection under the supervision of a study nurse. They were given an instruction booklet, reinjection calendar and three units to take home.	Clinic administration of DMPA-IM. Women who opted for DMPA-IM received their first injection from a study nurse, were instructed to return in 3 months and were given an appointment card.
Cover *et al* 14	Dakar and Thiés regions of Senegal. Women aged 18–45 years old attending participating health facilities for routine FP visits who expressed an interest in using injectable contraception (whether new, continuing or past injectable users). Mean age: (I) 26.9 (SD: 6.4); (C) 26.5 (SD: 6.2).	Controlled cohort study. 1299 participants (650 self-administration, 649 provider administration). 12-month follow-up.	Self-administration of DMPA-SC. Women were trained individually and their self-injection technique was evaluated for competency. Those judged competent were given three DMPA-SC units, an instruction booklet and a reinjection calendar. Those not competent were asked to return at the time of their second injection for refresher training, at which time their competency was reassessed and, if competent, they were given self-injection supplies.	Clinic administration of DMPA-IM. The DMPA-IM group received injections at the clinic and were given appointment cards to return for future injections.
Kohn *et al* 5	Texas and New Jersey, USA. Women ages 15–44 requesting DMPA, including method initiators and continuers. Age range: 16–44 (mean: 26 years).	Randomised controlled trial. 400 participants (200 self-administration, 200 provider administration). 12-month follow-up.	Self-administration of DMPA-SC. Women were taught to self-inject using printed instructions based on the drug packaging insert. If willing, participants then self-administered DMPA-SC under staff supervision. Those who correctly self-administered received three additional doses of DMPA-SC, a self-administration kit (including alcohol swabs, cotton pads, bandages, mini sharps disposal container) and printed self-administration instructions for the subsequent three injections, along with a calendar showing the appropriate injection dates. Participants received a reminder email and/or text message 2 weeks before each injection was due.	Clinic administration of DMPA-SC. Participants were administered DMPA-SC by qualified clinic personnel. Participants received a reminder email and/or text message 2 weeks before each injection was due.

C, control; DMPA, depot medroxyprogesterone acetate; DMPA-IM, DMPA intramuscular product; DMPA-SC, DMPA subcutaneous product; I, intervention.

Three studies were RCTs^5 11 12^; the other three studies were controlled cohort studies where women self-selected into the self-administration or provider administration study arms.^13–15^ The RCTs were generally found to have low risk of bias (table 2). However, there was high risk of performance and detection bias due to non-blinding, as it is impossible to blind participants and personnel to self-administration versus provider administration. The controlled cohort studies all compared two study groups and followed women over time (table 3). One controlled cohort study reported no statistically significant differences between study groups in measured baseline variables,^13^ while the other two conducted multivariate analyses to control for potential confounders after finding baseline differences between groups.^14 15^

**Table 2:** Cochrane risk of bias tool for randomised controlled trials

	Entry	Judgement	Support for judgement
Beasley *et al* 11	Random sequence generation (selection bias)	Low risk	’We stratified participants based on never, current, or past use of DMPA and randomized them to self or clinic administration. The sequence for the 2:1 (self vs clinic administration) treatment allocation was determined using a computerized random-number generator in blocks of six. An investigator not involved with participant contact generated the allocation schedule, which was concealed until after informed consent’.
	Allocation concealment (selection bias)	Low risk	’Group assignments for each stratum were placed in sequentially numbered opaque envelopes. After informed consent and screening were completed, the next envelope in the sequence was opened, and participants were enrolled by the study coordinator’.
	Blinding of participants and personnel (performance bias)	High risk	Blinding not possible given the nature of the intervention (for both participants and personnel) and may have affected outcomes of continuation.
	Blinding of outcome assessment (detection bias)	Low risk	No blinding of outcome assessment, but the outcome measurement is not likely to be influenced by lack of blinding (particularly with Medroxyprogesterone (MPA) levels).
	Incomplete outcome data addressed (attrition bias)	Low risk	Missing outcome data relatively balanced in numbers across intervention groups (10 of 86 in self-administration arm and 7 of 46 in clinic administration arm). While reasons for missing outcome data may be different across groups, the assumption that missing data indicated lack of continuation for both groups is strong.
	Selective reporting (reporting bias)	Uncertain risk	No protocol available (not described in paper or found on ClinicalTrials.gov). Insufficient information to permit judgement of ‘Low risk’ or ‘High risk’.
	Other bias	Low risk	The study appears to be free of other sources of bias.
Burke *et al* 12	Random sequence generation (selection bias)	Low risk	’Participants were randomly assigned (1:1) to receive DMPA-SC administered by a family planning provider or to be trained to self-inject DMPA-SC in accordance with a computer-generated block randomisation schedule with block sizes of four, six, and eight and stratification by study site’.
	Allocation concealment (selection bias)	Low risk	’Allocation concealment was achieved with sequentially numbered opaque envelopes’.
	Blinding of participants and personnel (performance bias)	High risk	Blinding not possible given the nature of the intervention (for both participants and personnel) and may have affected outcomes of continuation.
	Blinding of outcome assessment (detection bias)	High risk	No blinding of outcome assessment, and outcomes of continuation and adverse events/side effects may have been affected by lack of blinding. However, the statistical team remained masked until key decisions for the primary analysis were made.
	Incomplete outcome data addressed (attrition bias)	Low risk	No missing outcome data.
	Selective reporting (reporting bias)	Low risk	The study protocol is available, and all of the study’s prespecified (primary and secondary) outcomes that are of interest in the review have been reported in the prespecified way.
	Other bias	Low risk	The study appears to be free of other sources of bias.
Kohn *et al* 5	Random sequence generation (selection bias)	Low risk	’The sequence for the 1:1 (self vs clinic) treatment allocation was determined using a random number generator in blocks of six’.
	Allocation concealment (selection bias)	Low risk	’individual assignments were placed in sequentially numbered opaque envelopes. Following screening and informed consent, study staff enrolled each willing participant and opened the next envelope in the sequence’.
	Blinding of participants and personnel (performance bias)	High risk	Blinding not possible given the nature of the intervention (for both participants and personnel) and may have affected outcomes of continuation.
	Blinding of outcome assessment (detection bias)	High risk	No blinding of outcome assessment, and outcomes of continuation may have been affected by lack of blinding.
	Incomplete outcome data addressed (attrition bias)	Low risk	No missing outcome data.
	Selective reporting (reporting bias)	Low risk	The study protocol is available, and all of the study’s prespecified (primary and secondary) outcomes that are of interest in the review have been reported in the prespecified way.
	Other bias	Low risk	The study appears to be free of other sources of bias.

DMPA, depot medroxyprogesterone acetate; DMPA-SC, DMPA subcutaneous product; MPA, Medroxyprogesterone.

**Table 3:** Evidence Project risk of bias tool for non-randomised studies (all controlled cohort studies)

Citation	Study design includes pre/post intervention data	Study design includes control or comparison group	Study design includes cohort	Comparison groups equivalent at baseline on sociodemographics	Comparison groups equivalent at baseline on outcome measures	Participants randomly selected for assessment	Participants randomly allocated to the intervention	Control for potential confounders	Follow-up rate ≥75%
Cameron *et al*, 2011	No	Yes	Yes	Yes	NA	No	No*	No	No
Cover *et al* 15	No	Yes	Yes	No	NA	No	No*	Yes	Yes
Cover *et al* 14	No	Yes	Yes	No	NA	No	No*	Yes	Yes

*Women self-selected into intervention and control groups.

NA, not available.

The results from each study are presented in table 4, and meta-analysis summary results are presented in table 5. There was no evidence of significant heterogeneity in any meta-analyses. We did not use funnel plots to assess publication bias, as originally planned, due to the small number of included studies.

**Table 4:** Study outcomes

Citation	Results
Beasley *et al* 11	Continuation DMPA use at 1 year: (I) 71% (61/86); (C) 63% (29/46), p=0.47 Uninterrupted DMPA use at 1 year: (I) 47%; (C) 48%, p=0.70 Median number of days between the fourth and fifth injections: (I) 84 days (CI 84 to 89); (C) 84 days (CI 70 to 90), p=0.38 MPA level pg/mL among DMPA users at 12 months: (I) median: 640.8, mean: 695.8±318.5 pg/mL; (C) median: 641.0, mean: 686.2±309.6 pg/mL, p=0.85
Burke *et al* 12	Pregnancy: (I) 3/322; (C) 4/290, p=0.71. Side effects or adverse events Adverse events deemed potentially treatment-related: (I) 10/364 women (20 events); (C) 17/367 women (28 events) Serious adverse deemed potentially treatment-related: (I) 1/364 women (2 events); (C) 0/367 women (0 event). Any side effects Month 3: (I) 91/355 (26%); (C) 110/342 (32%) Month 6: (I) 55/324 (17%); (C) 56/254 (22%) Month 9: (I) 41/306 (13%); (C) 38/213 (18%) Abdominal pain, nausea, or vomiting Month 3: (I) 40/91 (44%); (C) 54/110 (49%) Month 6: (I) 27/55 (49%); (C) 25/56 (45%) Month 9: (I) 19/41 (46%); (C) 12/38 (32%) Irregular or heavy bleeding Month 3: (I) 33/91 (36%); (C) 48/110 (44%) Month 6: (I) 9/55 (16%); (C) 14/56 (25%) Month 9: (I) 7/41 (17%); (C) 12/38 (32%) Headaches Month 3: (I) 29/91 (32%); (C) 48/110 (44%) Month 6: (I) 17/55 (31%); (C) 19/56 (34%) Month 9: (I) 13/41 (32%); (C) 14/38 (37%) Injection site pain or irritation Month 3: (I) 38/91 (42%); (C) 27/110 (25%) Month 6: (I) 19/55 (35%); (C) 13/56 (23%) Month 9: (I) 16/41 (39%); (C) 5/38 (13%) Amenorrhoea Month 3: (I) 32/91 (35%); (C) 32/110 (29%) Month 6: (I) 28/55 (51%); (C) 22/56 (39%) Month 9: (I) 24/41 (59%); (C) 14/38 (37%) Backaches Month 3: (I) 27/91 (30%); (C) 33/110 (30%) Month 6: (I) 20/55 (36%); (C) 21/56 (38%) Month 9: (I) 16/41 (39%); (C) 17/38 (45%) Other aches or pains Month 3: (I) 26/91 (29%); (C) 20/110 (18%) Month 6: (I) 13/55 (24%); (C) 16/56 (29%) Month 9: (I) 8/41 (20%); (C) 8/38 (21%) Decreased libido Month 3: (I) 15/91 (16%); (C) 15/110 (14%) Month 6: (I) 9/55 (16%); (C) 9/56 (16%) Month 9: (I) 5/41 (12%); (C) 11/38 (29%) Weight changes Month 3: (I) 5/91 (5%); (C) 9/110 (8%) Month 6: (I) 7/55 (13%); (C) 8/56 (14%) Month 9: (I) 1/41 (2%); (C) 11/38 (29%)
	Continuation DMPA-SC continuation at 1 year: (I) 73% (256/364); (C) 45% (157/367), log-rank p<0.0001 Incidence of discontinuation: (I) 8.2 per 100 injection cycles (6.7–10.0); (C) 20.6 per 100 injection cycles (17.9–23.7), IRR 0.40, 95% CI 0.31 to 0.51, p<0.0001 Cumulative number of discontinuations/number at risk Month 0: (I) 1/364; (C) 0/367 Month 3: (I) 49/363; (C) 117/366 Month 6: (I) 79/309; (C) 165/245 Month 9: (I) 99/278; (C) 199/194 Sensitivity analysis with more lenient definition of continuation at 1 year: (I) 84%; (C) 53%, p<0.0001 Sensitivity analysis with more lenient definition of incidence of discontinuation: (I) 4.3 per 100 injection cycles (3.3–5.6); (C) 16.2 per 100 injection cycles (13.9–18.9), Interrater reliability (IRR) 0.27, 95% CI 0.19 to 0.36
Cameron *et al*, 2011	Continuation 12-month discontinuation rate: (I) 7/58 (12%), 95% CI 13% to 33%; (C) 14/64 (22%), 95% CI 6% to 23%, p=0.23 Side effects or adverse events Amenorrhoea at 1 month: (I) 93% (52/56); (C) 90% (42/48) Amenorrhoea at 12 months: (I) 96% (49/51); (C) 90% (34/39)
Cover *et al* 15	Pregnancy: (I) 3/561; (C) 2/600, not significant Continuation 12-month continuation cumulative probability: (I) 81%, 95% CI 78% to 84%; (C) 65%, 95% CI 61% to 69% Sensitivity analysis (data not shown) with those lost to follow-up excluded from analysis also found significantly greater probability of continuation in the self-injection group. Multivariate analysis of 12-month discontinuation Main effects model: HR 0.54 (0.44–0.68), p=0.00 Interaction model (including age): HR 0.75 (0.56–0.99), p=0.05 Side effects or adverse events Reported side effects After first injection: (I) 161/539 (29.9%); (C) 197/580 (34.0%), p>0.05 After second injection: (I) 117/497 (23.5%); (C) 135/489 (27.6%), p>0.05 After third injection: (I) 88/473 (18.6%); (C) 98/432 (22.7%), p>0.05 Sought advice for side effects After first injection: (I) 48/539 (8.9%); (C) 57/580 (9.8%), p>0.05 After second injection: (I) 33/497 (6.6%); (C) 47/489 (9.6%), p>0.05 After third injection: (I) 35/473 (7.4%); (C) 36/432 (8.3%), p>0.05 Reported ISRs After first injection: (I) 33/539 (6.1%); (C) 8/580 (1.4%), p<0.05 After second injection: (I) 25/497 (5.0%); (C) 8/489 (1.6%), p<0.05 After third injection: (I) 38/473 (8.0%); (C) 5/432 (1.2%), p<0.05 Sought advice for ISR: After first injection: (I) 0/539 (0%); (C) 2/580 (0.3%), p>0.05 After second injection: (I) 2/497 (0.4%); (C) 0/489 (0%), p>0.05 After third injection: (I) 3/473 (0.6%); (C) 2/432 (0.5%), p>0.05
Cover *et al* 14	Pregnancy: (I) 0/650; (C) 1/649 Continuation 12-month continuation rate: (I) 80.2%; (C) 70.4%, p<0.001 Multivariate analysis of 12-month discontinuation: adjusted HR 0.72 (0.56–0.93), p=0.00 Side effects or adverse events Serious adverse events No serious adverse events were reported in either group. Experienced side effects After first injection: (I) 195/649 (30.1%); (C) 227/642 (35.4%), p=0.04 After second injection: (I) 130/615 (21.1%); (C) 155/598 (25/9%), p=0.05 After third injection: (I) 102/588 (17.4%); (C) 125/559 (22.4%), p=0.03 Sought treatment for side effects After first injection: (I) 18/195 (9.2%); (C) 50/227 (22.0%), p=0.00 After second injection: (I) 17/130 (13.1%); (C) 32/155 (20.6%), p=0.09 After third injection: (I) 16/102 (15.7%); (C) 28/125 (22.4%), p=0.20 Experienced ISRs After first injection: (I) 89/649 (13.7%); (C) 63/642 (9.8%), p=0.03 After second injection: (I) 52/615 (8.5%); (C) 55/598 (9.2%), p=0.65 After third injection: (I) 24/588 (4.9%); (C) 30/559 (5.4%), p=0.74 Sought treatment for ISR After first injection: (I) 0/89 (0%); (C) 0/63 (0%), p= – After second injection: (I) 0/52 (0%); (C) 0/55 (0%), p= – After third injection: (I) 0/29 (0%); (C) 1/30 (3.3%), p=0.32
Kohn *et al* 5	Pregnancy: (I) 0/200; (C) 3/200 Continuation 1-year continuous DMPA use: (I) 69%; (C) 54%, RD: 15%, 95% CI 5% to 26%, p=0.005. 6-month continuous DMPA use: (I) 87%; (C) 69%, RD: 18%, 95% CI 9% to 27%, p<0.001. Stratified analysis by age: ≤19 years: 67%, ≥20 years: 60%, p=0.46. Relaxed definition of continuation (received four shots during the study period, any dose intervals): (I) 78%; (C) 64%, p=0.008. Per-protocol sensitivity analysis removing women who were assigned to self-injection but had a nurse administer the first injection showed a consistent magnitude and direction of effect. As-treated analysis reassigning self-administration subjects who crossed over to clinic group: (I) 68%; (C) 54%, RD: 14%, 95% CI 4% to 25%. Sensitivity analysis classifying those who withdrew or were lost to follow-up as discontinued found a similar effect in direction and magnitude.

C, control;DMPA, depot medroxyprogesterone acetate; DMPA-SC, DMPA subcutaneous product; I, intervention;IRR, Interrater reliability; ISR, injection site reaction; RD, risk difference.

**Table 5:** Meta-analysis results summary

Outcome	Type of studies	Studies (n)	RR*	Lower limit	Upper limit	P value for RR	Q-value	P value for Q statistic	I-squared
Continuation of injectable contraception	RCTs	3	1.272	1.163	1.391	0.000	0.984	0.611	0.000
	Observational studies	3	1.178	1.100	1.262	0.000	3.770	0.152	46.951
Pregnancy	RCTs	2	0.582	0.153	2.217	0.428	0.580	0.446	0.000
	Observational studies	2	1.107	0.233	5.261	0.899	0.700	0.403	0.000

*Relative risk (risk ratio) comparing self-administration with provider administration.

RCTs, randomised controlled trials.

### Continuation of injectable contraception

All six studies measured continuation (or discontinuation) of injectable contraception. One study measured continuation through Medroxyprogesterone (MPA) levels,^11^ while the others assessed continuation through self-report.^5 12–15^

**Figure 2:**
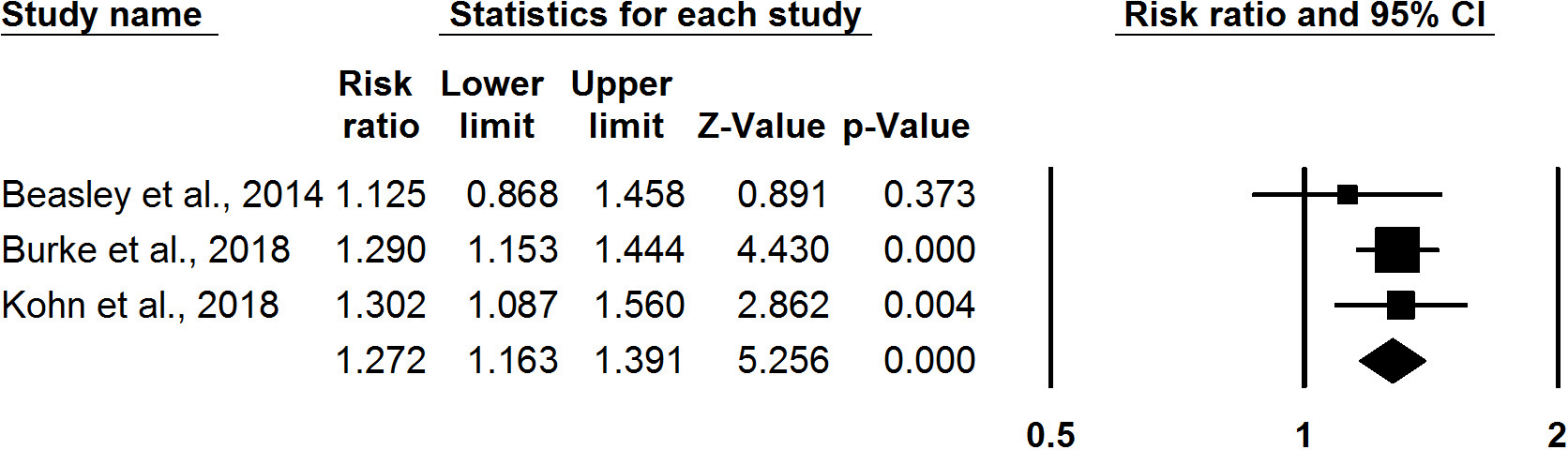
Meta-analysis of contraceptive continuation: results from randomised controlled trials.

**Figure 3:**
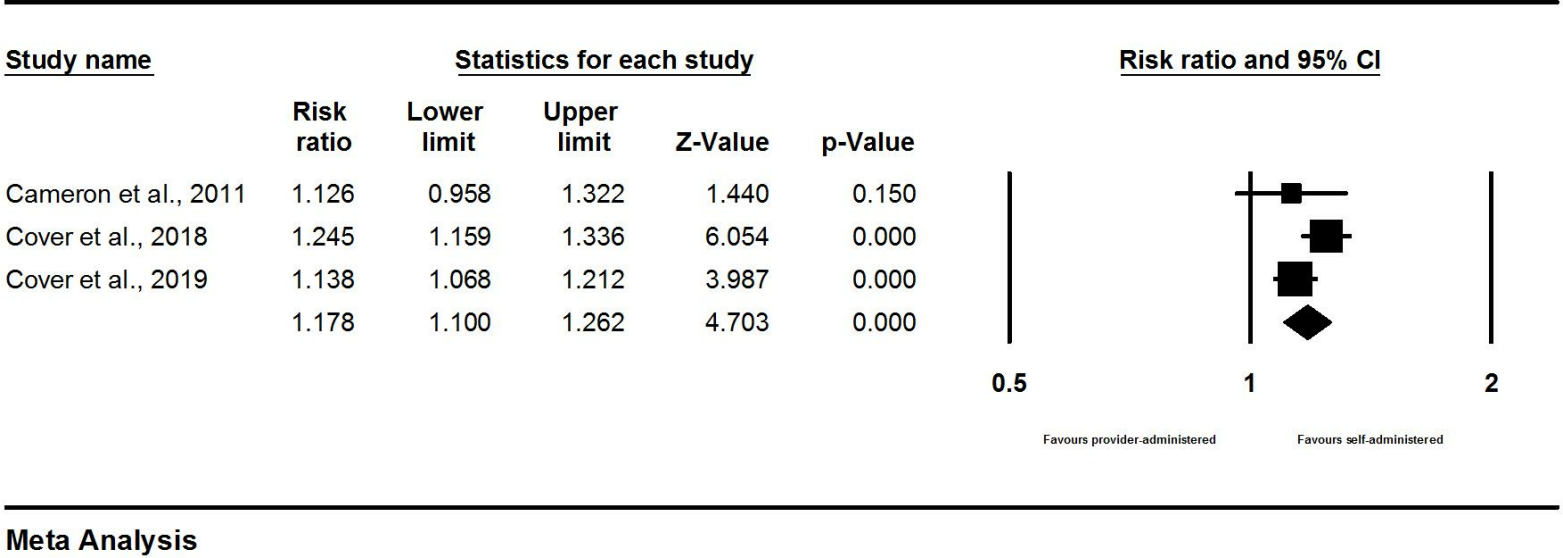
Meta-analysis of contraceptive continuation: results from observational studies.

Among three RCTs, meta-analysis found that self-administration was associated with greater continuation at 12 months compared with provider administration (RR: 1.27, 95% CI 1.16 to 1.39) (figure 2). Meta-analysis results from the three observational studies found a remarkably consistent pattern with the RCTs: self-administration was associated with increased likelihood of continuation at 12 months compared with provider administration (RR: 1.18, 95% CI 1.10 to 1.26) (figure 3).

### Pregnancy

Four studies reported participant pregnancies.^5 12 14 15^ In all four studies, there were few reported pregnancies (<5) in both self-administration and provider administration groups (table 4). In meta-analysis, participants in the self-administration study arms were no more likely to become pregnant than those in the provider administration arms in either RCTs or observational studies (table 5); however, the small number of pregnancy events in these trials means that the actual effect size estimates for this outcome should be interpreted with caution.

### Side effects or adverse events

Four studies reported side effects or adverse events.^12–15^ One RCT reported side effects and adverse events.^12^ Overall, there were no significant differences in the proportions of women reporting side effects, adverse events and serious adverse events between the self-administered and provider-administered groups. For serious adverse events, there was just one event—a case of severe back pain in the provider-administered group. There were, however, some differences in specific side effects at specific time points; specifically, women in the self-administration arm were more likely to report injection site pain or irritation at 3 months and at 9 months.

Two large controlled cohort studies found that more women who self-injected reported experiencing an injection site reaction at one or more follow-up visits; there were no differences in the reported side effects overall in Uganda, and in Senegal there were fewer reported side effects among women in the self-administered arm compared with the provider-administered arm at each follow-up, although the type of injectable used differed across arms.^14 15^ Finally, one controlled cohort study found similar rates of amenorrhoea at 1 and 6 months in the self-administration and provider administration groups.^13^

### Social harms

No studies presented data on social harms in published articles. However, personal communication from the principal investigator of one RCT revealed that staff were trained to report social harms throughout the trial, and there was a form available to record such harms, but no social harms were reported through these mechanisms during the trial.^12^

### Other outcomes

No studies reported measuring self-efficacy or knowledge and empowerment. We also found no studies that specifically reported initial uptake of hormonal contraception; while some studies did include initial users, analyses were not stratified by initial versus continuing users.

### Stratifications

Regarding the type of support offered to self-administration, two studies from Scotland and the USA provided text messages and/or emails to remind participants of their next injection; one study provided this only to participants in the self-injection group,^13^ while the other study provided reminders to both study arms.^5^ All other studies provided some sort of calendar to remind participants of their reinjection window. There were no clear differences in outcomes based on reminder usage.

Regarding age, only one RCT from the USA included young women under the age of 18^5^; this study found that continuation was similar by age group (67% in ≤19 years vs 60% in ≥20 years, p=0.46), but the results for the association between self-injection and continuation were not stratified by age. In the two controlled cohort studies from Uganda and Senegal, age was included in multivariable analyses of continuation.^14 15^ In Uganda, the interaction term showed that the effect of injection group varied by age: for those 25 and older, self-injecting reduced the risk of discontinuation by 25%, while for youth ages 18–24, self-injecting reduced the risk by 40%.^15^ However, the interaction effect was non-significant in Senegal, suggesting no difference in the effect of self-administration by age (personal communication from the study authors).^14^ These same two studies included measures of household assets in multivariable analyses of overall continuation for both study groups, but not separately for self-administration and provider administration groups.^14 15^

Finally, regarding high-income versus low-income or middle-income countries, the two studies that showed no statistically significant difference in continuation across study arms were from high-income countries.^11 13^ However, these were also the two studies with the smallest number of participants, and both showed effect estimates that indicated increased continuation with self-administration, so their lack of significant results may have been due to inadequate power rather than high-income setting.

## Discussion

A small but growing evidence base suggests that self-administration of DMPA-SC can lead to equal or improved contraceptive continuation rates compared with provider administration. These findings are consistent in RCTs and observational studies. Further, there is no evidence to suggest that this benefit comes with increased pregnancy rates or safety concerns. While two studies included in the review did find higher rates of injection site reactions among women who self-injected DMPA-SC, this may be due to the type of injection (subcutaneous) rather than the self-administration, as both studies compared self-administration of DMPA-SC with provider administration of DMPA-IM. A recent systematic review of the safety of DMPA-SC found that users ‘may experience injection site reactions more frequently, but these are rare, typically mild to moderate in severity and generally resolve without further intervention’.^3^

Our findings are limited by the relatively small evidence base identified, which is likely due to the fact that subcutaneous injection of hormonal contraception is relatively new, and thus many of the studies we identified were from just the past few years. Our findings may have also been limited by our reliance on peer-reviewed studies. However, review of trial registries did not identify any completed trials that remained unpublished, and results across studies were generally quite consistent. Given the small number of included studies and the general consistency of findings across studies, it was difficult to discern meaningful differences across groups in our preplanned stratified analyses.

The small number of pregnancy events recorded in the included studies means that the actual effect size estimates for this outcome should be interpreted with caution, especially given that our meta-analyses relied on a continuity correction which may lead to biased estimates.^17^ However, while the exact effect size estimate may be questionable, we feel confident in the general conclusion that there was no significant difference across study arms. We also found no studies that reported measuring self-efficacy, knowledge and empowerment, or social harms. Future studies could consider including these outcomes to more broadly measure potentially important aspects of contraceptive self-injection programmes.

A number of countries are currently considering contraceptive self-injection programmes. These programmes should follow existing WHO guidance which notes that self-injection should be provided only ‘in contexts where mechanisms to provide the woman with appropriate information and training exist, referral linkages to health care providers are strong, and where monitoring and follow-up can be ensured’.^18^ Critical programmatic issues, such as equipping women with the necessary knowledge and materials to keep and dispose of sharps and accurately determine their reinjection window, must be addressed in any programmatic roll-out, and also balanced with the important considerations of convenience and discretion for this self-care intervention.

We also emphasise the importance of offering self-injection of hormonal contraception as part of a comprehensive family planning programme offering a range of contraceptive choices. Recent evidence, although mixed, suggests DMPA may be associated with an increased risk of HIV acquisition^19^; based on this evidence, the WHO recently changed their guidance for women who are at high individual risk for HIV acquisition, moving progestogen-only injectable contraceptives from a category 1 (no restriction for use) to a category 2 (benefits outweigh risks).^20^ Results from the Evidence for Contraceptive Options and HIV Outcomes trial—the first randomised trial to examine the effect of different contraceptive methods, including DMPA-IM, on HIV incidence—are expected in mid-2019, which will provide stronger information on whether DMPA is associated with a greater risk of HIV acquisition compared with the implant and copper intrauterine device.^21^ On the release of these results, WHO will convene a Guideline Development Group to review the updated body of evidence and will issue guidance following WHO’s requirements for guideline development^22^ through an expedited process. Programmes can consider using tools such as Planning4Outcomes to estimate the potential impact of a change in injectable contraceptive use on both pregnancy and HIV outcomes.^23^ This issue underscores the importance of having a contraceptive method mix available in national family planning programmes, so that women have the fullest range of contraceptive choices possible. As programmes consider adding self-injection contraception options, these should expand, rather than replace, contraceptives in the available method mix.

In conclusion, self-injection of hormonal contraception, specifically DMPA-SC, is a promising approach to increasing contraceptive use. This information has been used to inform the development of WHO recommendations for self-care interventions for SRHR in relation to self-administration of injectable contraception. The benefits and harms of the intervention found in the present review, along with values and preferences, resource use, human rights, and feasibility issues, will shape the recommendation. Additional research into outcomes such as social harms and self-efficacy/empowerment should be done to address the gaps identified.
